# Impacts of climate change on infestations of Dubas bug (*Ommatissus lybicus* Bergevin) on date palms in Oman

**DOI:** 10.7717/peerj.5545

**Published:** 2018-09-05

**Authors:** Farzin Shabani, Lalit Kumar, Rashid Hamdan Saif al Shidi

**Affiliations:** School of Environmental and Rural Science, University of New England, Armidale, NSW, Australia

**Keywords:** Dubas bug, Climate change, *Ommatissus lybicus* Bergevin, Date palms

## Abstract

Climate change has determined shifts in distributions of species and is likely to affect species in the future. Our study aimed to (i) demonstrate the linkage between spatial climatic variability and the current and historical Dubas bug (*Ommatissus lybicus* Bergevin) distribution in Oman and (ii) model areas becoming highly suitable for the pest in the future. The Dubas bug is a pest of date palm trees that can reduce the crop yield by 50% under future climate scenarios in Oman. Projections were made in three species distribution models; generalized linear model, maximum entropy, boosted regression tree using of four global circulation models (GCMs) (a) HadGEM2, (b) CCSM4, (c) MIROC5 and (d) HadGEM2-AO, under four representative concentration pathways (2.6, 4.5, 6.0 and 8.5) for the years 2050 and 2070. We utilized the most commonly used threshold of maximum sensitivity + specificity for classifying outputs. Results indicated that northern Oman is currently at great risk of Dubas bug infestations (highly suitable climatically) and the infestations level will remain high in 2050 and 2070. Other non-climatic integrated pest management methods may be greater value than climatic parameters for monitoring infestation levels, and may provide more effective strategies to manage Dubas bug infestations in Oman. This would ensure the continuing competitiveness of Oman in the global date fruit market and preserve national yields.

## Introduction

*Ommatissus lybicus*, formerly classified as the “lybicus” variety of *O. binotatus* and commonly described as the “Old World date bug” or “Dubas bug”, was elevated taxonomically to the status of species as *O. lybicus* Bergevin (Asche & Wilson, 1989). Renowned for its voluminous production of honeydew (Dowson, 1936), the Dubas bug attacks date palms in the Mediterranean, which appear to be its sole host. Adult females measure between five and six mm in length, vary from yellowish brown to greenish in color and exhibit pairs of black dots on the base of the frons and pronotum, as well as frequently on the top of the head and the seventh and eighth segments of the abdomen. The wings are clear, with visible blood vessels concentrated at the apex, a distinctive characteristic of the family. Males are slightly smaller than females, with increased proportion of wingspan to body length, a narrower abdomen and absence of abdominal dots (Asche & Wilson, 1989; Howard et al., 2001).

**Figure 1:**
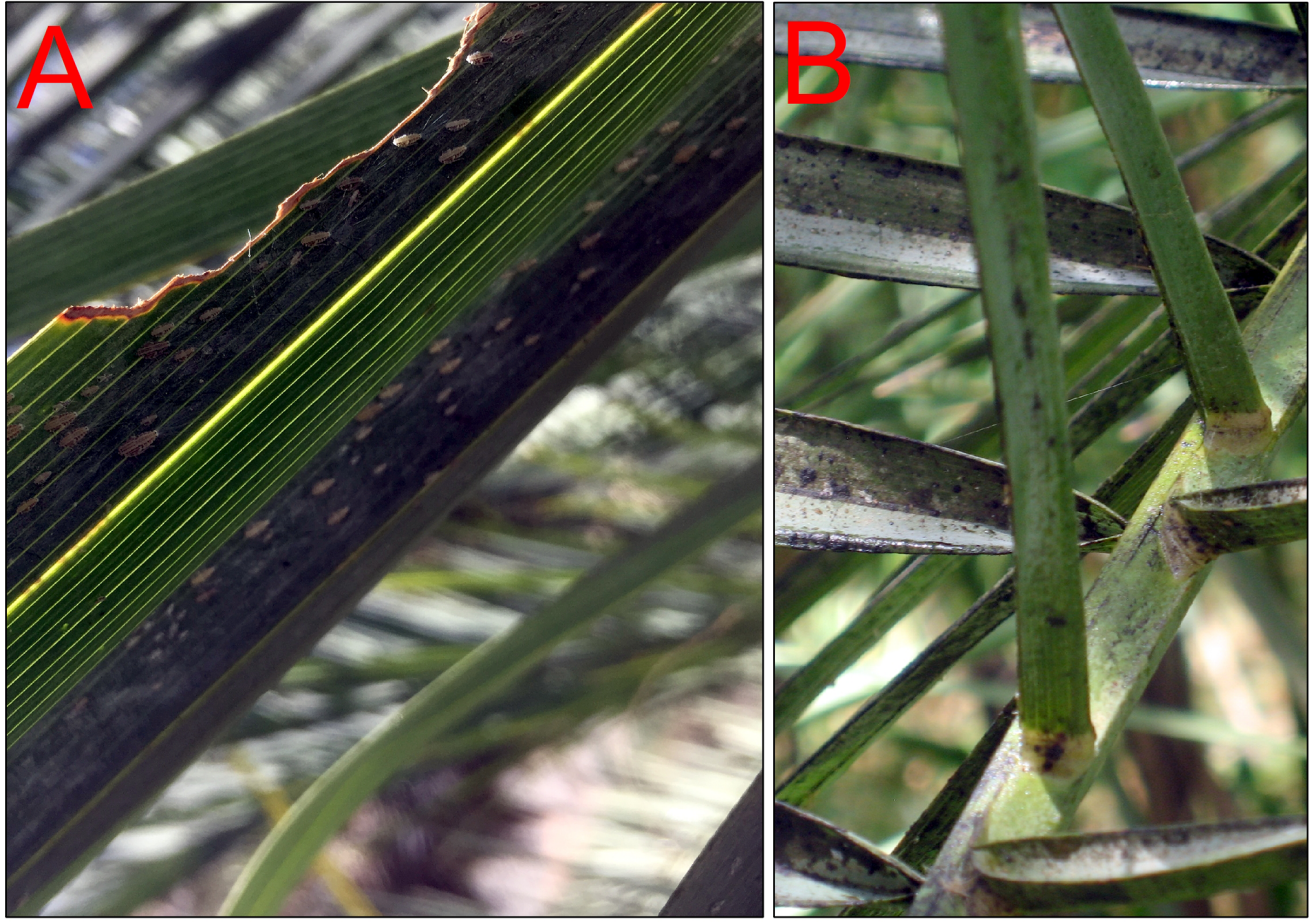
(A) Dubas adults and nymphs suck sap and coat leaf surfaces. (B) Sooty mold development on honeydew coated leaves of date palms in Oman.

Date palm regions such as Iran and specially Oman suffer regular Dubas bug infestations, resulting in substantial losses (Abdullah, Lorca & Jansson, 2010; Blumberg, 2008). Over the last four decades, the species has become rated as the major date palm pest of Oman, in terms of area and severity of infestation and consequent losses (Mamoon, Wright & Dobson, 2016). Direct damage is caused by adults and nymphs feeding on the plant sap and covering the surfaces of leaves with honeydew (Fig. 1), leading to indirect damage through the progression of a sooty mold (Fig. 1) and a resultant reduction in yield and date quality (Howard et al., 2001). Research shows that direct damage occurs through the sucking of sap from leaflets and rachis during spring and autumn (Kinaway & Al-Siyabi, 2012). Indirect damage is exemplified in the deterioration of date palm fruits, including the fruit of trees planted beneath infested palms, due to dust, dry leaflets and rot fungi attracted by the honey dew. Indirect damage is exacerbated by females depositing eggs beneath the surface of the biaxial frond, restricting photosynthesis. Gassouma (2004) has documented that a heavy Dubas bug infestation can reduce yield by as much as 50%. A heavy infestation implies thousands of the bugs per frond (Dowson, 1936), and generally occurs on mature date palms, probably due to the greater protection afforded by larger trees (Klein & Venezian, 1985). Higher humidity levels, denser plant spacing and greater shading promote Dubas population growth, which can persist through dust-storms and lengthy hot, dry periods (Howard et al., 2001). The Dubas population endured mild Mediterranean winters but perished in the severe Israeli winter nights with the freezing temperatures of 1982/1983.

Climate change presents a major threat to the global biodiversity and ecosystems, on which humans are dependent (Rosenzweig et al., 2008). There is strong evidence that climate change has already caused changes to distributions of species, and will continue to do so (Paterson et al., 2017; Shabani, Kumar & Taylor, 2012; Shabani, Kumar & Esmaeili, 2015; Shabani et al., 2013; Shabani et al., 2018; VanDerWal et al., 2013), through alterations in phenology (Thackeray et al., 2010), physiology (Rosenzweig et al., 2008), morphology (Walsh et al., 2016), demography and community composition (Bellard et al., 2012) and the nature of ecological interactions (Parmesan, 2006). Extinctions at species level have been reported (Sinervo et al., 2010), with rates of extinction projected to accelerate as climate change intensifies (Urban, 2015). To minimize biodiversity losses, conservationists, resource managers and decision-makers must adapt environmental policies and management practices toward ameliorating the impact of climate change (Brooke, 2008).

Species distribution models (SDMs) allow the incorporation of climate change scenarios into modeling, thus providing information on potential future species distributions. Such analyses can highlight specific new areas that may in the future be at risk of invasion, as well as identifying the important regions of biodiversity that may be affected. Mapping potential future distributions can inform the strategic planning of biosecurity agencies, prioritizing areas that should be targeted for eradication and determining those areas’ containment tactics would be more cost-effective. Such models are alternatively described as bioclimatic or ecological niche models (Fitzpatrick et al., 2007). On this matter, a range of computer-based systems have been developed, designed for the modeling of current or future distributions of the species. Examples of the most common of these systems are CLIMEX (a mechanistic model), HABITAT, maximum entropy (MaxEnt), boosted regression trees (BRT), random forests, generalized linear model (GLM) and BIOCLIM (correlative models). The key component of the ecological niche modeling approaches is estimation or characterization of species’ distributions in ecological space, which can then be useful in understanding their potential distributions in geographic space (Peterson, 2006). A greater capacity to model the impact of climate change on the distributions of species will be invaluable toward this end (Da Silva et al., 2017; Dawson et al., 2011; Peterson et al., 2017; Ramirez-Cabral, Kumar & Shabani, 2017; Shabani, Kumar & Ahmadi, 2017; Soberon & Peterson, 2005).

Despite the potential devastation of the Dubas bug, this appears to be the first published study that focuses on projecting the risk levels of colonization. Estimating future distributions of the species under a variety of climate scenarios is of interest for regions cultivating date palm, including Oman. Our study employed four global circulation models (GCMs), (a) HadGEM2, (b) CCSM4, (c) MIROC5 and (d) HadGEM2-AO, under four representative concentration pathways (RCPs) of 2.6, 4.5, 6.0 and 8.5, for two time periods of 2050 and 2070 using GLM, MaxEnt, BRT. It should be noted that there are 19 (GCMs) in WorldClim database and in this study, we have selected four of them randomly. We believe that climate change may influence the potential future distribution of the Dubas bug and our analysis makes a positive contribution to the optimization of date palm production, management and control of the Dubas bug.

## Materials and Methods

### Data collection area and pest distribution records

**Figure 2:**
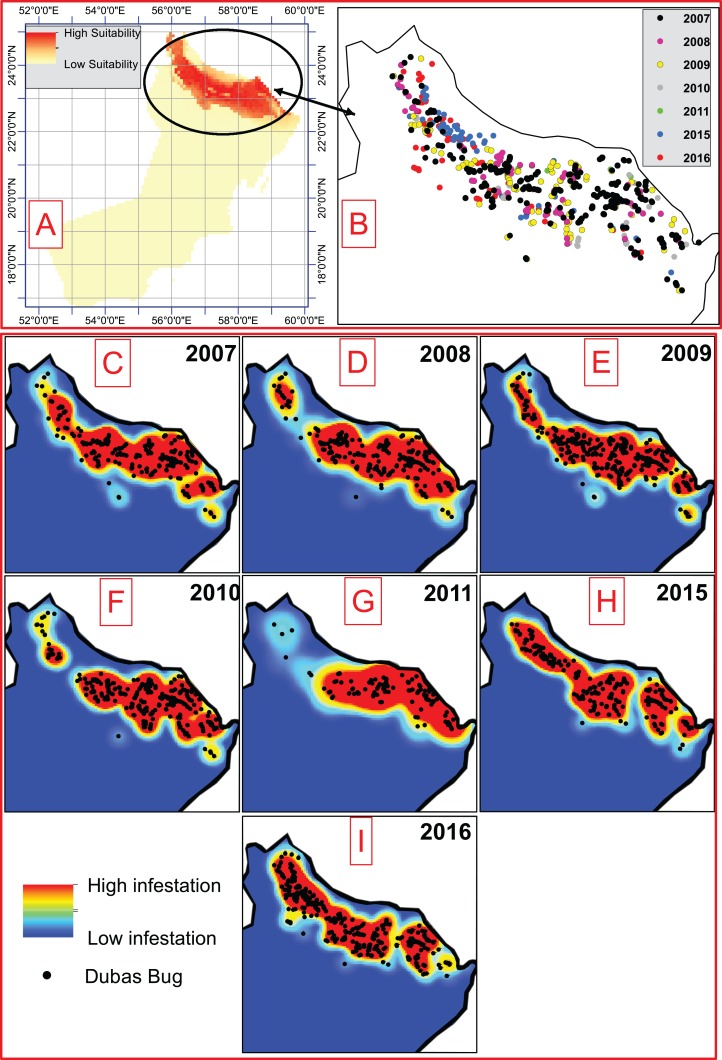
(A) Current climate suitability for Dubas bug. (B) Distribution data of the Dubas bug for of the periods 2007–2011, 2015 and 2016. (C–I) Annual hotspots indicating areas with high density of the pest for the period.

The data collection area covered 69 villages (300 locations) isolated from each other in the northern governorates of Oman where date palm is cultivated. The total area of Oman is about 310,000 km^2^; however, the data collection area was limited to the northern part of Oman (North 26°00′N, 56°00′E and Far East 22°00′N, 59°00′E) where date palm is cultivated as the main agricultural crop. Distribution data (1,708 occurrences) for the years 2007–2011 and 2015, was obtained from the Ministry of Agriculture of Oman. Our database was extended through field trips in 2016, illustrated in Fig. 2. Using the annual distribution data and linear Kriging interpolation method, we identified historical changes of hotspots (directions between sample points that reflect annual variations in the surface). A total of 20% of the records were reserved for model validation. The data collection between 2007 and 2011 was carried out by local experts who were working at Ministry of Agriculture of Oman, and then this project was stopped between 2011 and 2015 and then started again. It should be mentioned that the last field trip was undertaken by our group in 2015 and the data is included in this study. We should also highlight that there are some data available for the 2012–2014 period, but their accuracy is questionable and thus we excluded them from our analysis.

### Linkage between current spatial climatic variability and the Dubas bug distribution

The climate data for the year 2007, 2008, 2009, 2010, 2011, 2015 and 2016 were downloaded from the *National Environment Satellite, Data and Information* Service website (https://www7.ncdc.noaa.gov/CDO/cdo). Here, we selected the closest meteorological stations from the areas with the historical pest occurrence records. This data contains the daily means of temperature, minimum temperature and maximum temperature, wind speed, dew points, sea level pressure, station pressure, visibility, maximum sustained wind speed and total daily precipitation. In technical terms, the dew point is the temperature at which the water vapor in a sample of air at constant barometric pressure condenses into liquid water at the same rate at which it evaporates. The data was used to predict the difference in climate factors for all the infested locations for each year using the inverse distance weighted interpolation method in ArcMap. Then, the ordinary least squares (OLS) regression method was used to examine the global relationship and the geographic weight regression (GWR) was used to find the local spatial relationship between the infestation and the different climate factors for each year. The GWR statistical model considers the spatial variance of the variables and estimates the strength of their topographic relationships (Refer to Fotheringham, Crespo & Yao, 2015). The GWR model formulation is:

$${{\rm{y}}_i}={{\rm{\beta }}_{\rm{0}}}{\rm{(}}{u_i}{\rm{, }}{v_i}{\rm)} + \Sigma {{\rm{\beta }}_{\rm{1}}}{\rm{(}}{u_i}{\rm{, }}{v_i}{\rm{) }}{x_{ik}}+{{\rm{\varepsilon }}_i}$$

where *i* is the spatial point with coordinates (*u_i_*, *v_i_*). Accordingly, *y_i_* is the dependent variable, *x_ik_* is the *k*th independent variable, *ε_i_* is an error term for the *i*th point.

The first run of OLS showed a high variance inflation factor indicating a high level of redundancy among explanatory variables. The factors showing high redundancy were removed. The GWR was then run with the remaining factors.

### Calibration area

In this research calibration of the models was based on 75% of the occurrence dataset (training data) from 56°30′00″ to 59°00′00″, with the remaining 25% from 56°00′00″ to 56°30′00″ and 59°00′00″ to 59°30′00″ reserved for evaluating the performance of the models. We purposefully selected testing points in those regions (western and eastern Oman) that had geographic outliers. We believe that this gives a better basis for validation of the model compared to selecting test points from regions of high occurrence data.

### Distribution modeling approach

Biomod2 ensemble platform for species distribution modeling (Thuiller et al., 2009) was used to predict suitable pest habitat, in R environment v. 3.4.4 (R Development Core Team, 2017). The method enables the simultaneous processing of a number of modeling techniques to create a consensus, or “ensemble”, model (Araújo & New, 2007; Thuiller et al., 2009). We used three methods, MaxEnt, GLM and BRT, to form an integrated prediction of the pest preferred habitat with thirty replicates. The main motivation that led to use three different mechanistic models was that each of the models relies to some degree on parameterization against observational data, so they do not entirely avoid the novelty challenge and their predictions are not necessarily superior to empirical models (Fordham et al., 2018; Shabani, Kumar & Ahmadi, 2016). Additionally, it has been suggested that on the species distribution modeling studies, it may be safer to utilize several SDMs and ensemble the results (Shabani, Kumar & Ahmadi, 2016). In line with this matter, Elith, Kearney & Phillips (2010) documented that the area that has been accessible to the species of interest over relevant time periods represents the ideal area for model development, testing and comparison.

### Maximum entropy

The MaxEnt algorithm compares the interaction of variables on presence and background data to establish the probability distribution approximating uniformity, subject to the limitations of the spatial distributions observed and related environmental factors. This method of minimizing relative entropy between presence and background data optimizes the probability distribution representing MaxEnt (Phillips, Anderson & Schapire, 2006).

### Generalized linear model

In GLM, the iterative weighted linear regression technique was used to arrive at the estimated maximum likelihood of the parameters, with observations distributed in terms of an exponential family and systematic effects made linear by suitable transformation. For GLM, parametric functions were employed to link the variable of response to a combination of linear and quadratic explanatory variables. The GLMs were fitted with a standard polynomial approach together with an automatic stepwise model selection based on the Akaike information criterion (AIC).

### Boosted regression tree

Boosted regression tree uses two multiple regression tree algorithms (by a binary division of predictor space into rectangles, it relates predictor responses to establish expanses with the most homogeneous responses to predictors) and boosting (an added procedure, merging fitted trees for greater modeling accuracy). BRT was fitted using the “GBM” package (Ridgeway, 2006) in R environment v. 3.4.4 (R Development Core Team, 2017) with additional setting code recommended by Elith, Leathwick & Hastie (2008).

### Bioclim variables, background data and provision of weights for records of the species

Baseline climate was represented by the WorldClim current climate dataset of BIOCLIM variables (www.worldclim.org). Here, we used WorldClim data of Version 1.4 with 2.5 min resolution grids. WorldClim is a high-resolution climate average for the period 1961–1990, with global coverage and spanning the time period over which the majority of occurrence records were collected. Possible future climates at global scale incorporate four IPCC5 greenhouse gas concentration (GHC) trajectories, which differ in terms of GHC emission peaks. The main objective of this study was to utilize RCP of 2.6, 4.5, 6.0 and 8.5 for incorporation into the future climate scenarios in the model projections. Our data has 2.5 min spatial resolutions. To eliminate model complexity and screening explanatory variables, we used the jackknife analysis method and calculated the pairwise Pearson correlation matrix of the variables to select the more important variables showing low correlation (*R* < 0.7). We also checked the importance of variables through the correlation coefficient from Pearson correlation technique and the results were the same as jackknife outputs. Thus, bio7 (temperature annual range (°C)), bio8 (mean temperature of wettest quarter (°C)), bio9 (mean temperature of driest quarter (°C)), bio10 (mean temperature of warmest quarter (°C)), bio11 (mean temperature of coldest quarter (°C)), bio15 (precipitation seasonality) and bio16 (precipitation of wettest quarter (mm)) were selected. We note that in this study, clamping was not used and to address the likelihood that the background data would contain fewer records from localities of more recent colonization and those poorly sampled, we denoted greater importance to records with less geographic proximity. We took into account that without records measuring time expended and survey effort, it is impossible to distinguish between unsuitable and under-sampled areas and that the above-mentioned adjustments would unavoidably combine these two geographical categories. For calculation of the weighting surface, we divided the total of weighted records for each cell of the study area by the weighted number of terrestrial cells of the specific area, using the Gaussian kernel method with standard deviations of ArcGIS default values to eliminate the coastal edge effect. Thereafter, we adjusted the resulting grid to maximum 20 and minimum 1, to exclude extreme values. This weighting method, as advocated by Elith, Kearney & Phillips (2010), minimizes the bias toward records from highly sampled areas over those from less sampled areas. Background training data was generated using the kernel density layer and Hawths Tools extension (v3.27) (Beyer, 2004).

### Threshold

There are many methods of thresholds selection, including setting 0.5 as the threshold (default), which is widely used in ecology (Pearson et al., 2002), setting a specific level of sensitivity or specificity (e.g., 95%) as desired or deemed acceptable (Cantor et al., 1999) and setting thresholds to maximize the correlation between known and projected distributions. A further method identifies a value that maximizes points correctly classified; sensitivity plus specificity values; or Kappa, which incorporates both sensitivity and specificity (Guisan, Theurillat & Kienast, 1998). We chose the commonly used threshold of maximum sensitivity + specificity classifying outputs.

### Global circulation models

There are many different GCMs in WorldClim database and we have selected the four GCMs of HadGEM2, CCSM4, MIROC5 and HadGEM2-AO randomly. For example, HadGEM2-ES is a model from the Hadley Centre Global Environmental Model associated cycle of the fifth phase of the CMIP5 (Taylor, Stouffer & Meehl, 2012) which combines dynamic vegetation, ocean biology and atmospheric chemistry, inclusive of greenhouse gases emissions, aerosols, solar irradiance, ozone and others (Dike et al., 2015). HadGEM2-ES demonstrates a high climate sensitivity of approximately 4.68 °C for a doubling of CO_2_, placing it as one of the more efficient CMIP5 models (Andrews et al., 2012; Dike et al., 2015) and couples an atmospheric and ocean model more realistically simulating the uptake and retention of carbon dioxide at varying ocean depths (Heffernan, 2010).

The community climate system model (CCSM) is a coupled climate model used to simulate the climate system of earth. Four individual models are coupled to simulate simultaneously the planet’s atmosphere, oceans, land surfaces and sea-ice, using a single coupling component. The CCSM enables fundamental research into Earth’s past, present and future climate states of CESM1. The code base of CCSM4 has been frozen and future updates will use the code base of CESM1, of which CCSM4 is a subset. Although CESM1 supersedes CCSM4, all CCSM4 experiments may be run using the CESM1 code base (Dike et al., 2015). Detailed information on the utilized GCMs and their differences can be found in Lawrence et al. (2012), Watanabe et al. (2010) and Baek et al. (2013).

### Representative concentration pathways

Representative concentration pathway 2.6 assumes that the global warming will increase from 0.4 to 1.6 and 0.3 to 1.7 °C between the years 2046 and 2064 and 2081 to 2100, respectively. RCP 2.6 also assumes that the mean global sea level will increase from 0.17 to 0.32 and 0.26 to 0.55 m, over the same periods (Stocker et al., 2013).

Representative concentration pathway 4.5 assumes that the global warming will increase from 0.9 to 2.0 and 1.1 to 2.6 °C between the years 2046 and 2064 and 2081–2100, respectively. Mean global sea level will increase from 0.19 to 0.33 and 0.32 to 0.63 m over the same periods (Stocker et al., 2013).

Representative concentration pathway 6.0 assumes that the global warming will rise from 0.8 to 1.8 and 1.4 to 3.1 °C between the years 2046 and 2064 and 2081–2100, respectively. Mean global sea level will increase from 0.18 to 0.32 and 0.33 to 0.63 m over the same periods (Stocker et al., 2013).

Representative concentration pathway 8.5 assumes that the global warming will rise from 1.4 to 2.6 and 2.6 to 4.8 °C between the years 2046 and 2064 and 2081–2100, respectively. Mean global sea level will increase from 0.22 to 0.38 and 0.45 to 0.82 m over the same periods (Stocker et al., 2013).

In this study, we focused on all four RCPs 2.6, 4.5, 6.0 and 8.5 to identify areas possibly becoming highly suitable, unsuitable or areas with significant change compared to the pest current suitability regions. In addition, the results of this paper at a global scale are presented in the supplementary section.

### Model validation

Using training and testing pest presence data (#1196 and #512, respectively), validation was initially carried out using the area under curve (AUC) method. However, as highlighted by other studies (Jiménez-Valverde et al., 2013; Lobo, Jiménez-Valverde & Real, 2008; Peterson, Papeş & Soberón, 2008), AUC values can be misleading and unfairly denigrate the accuracy of the model (Fourcade et al., 2014). AUC is considered a reliable measure of discrimination ability but, when estimations are based only on presence data, has been seen to have limitations in attaining ecological realism in modelled distribution (Jiménez-Valverde, 2014; Lobo, Jiménez-Valverde & Real, 2008), we also introduced true skill statistic (TSS) for validating our model. TSS is independent of prevalence and equals ${{ad - bc} \over {\left( {a + c} \right)\left( {b + d} \right)}}$ where *a* signifies number of correctly predicted presence cells; *c* number of presence cells incorrectly predicted as absence cells; *b* number of absence cells incorrectly predicted as presence cells; and *d* number of correctly predicted absence cells. It is essential to recognize that across our models, sensitivity and specificity are mutually independent, as well as being independent of prevalence, which represents the proportion of total cells that recorded species presence. Allouche, Tsoar & Kadmon (2006) demonstrated that TSS represents an intuitive method of SDM predictive performance measurement transposed into presence–absence mapping. TSS produced results that are significantly correlated with those of the threshold-independent AUC assessment (Allouche, Tsoar & Kadmon, 2006). AUC and TSS values of our modeling were 0.94 and 0.89, respectively.

## Results

### Positive linkage between current spatial climatic variability and the Dubas bug distribution

Considering all examined climatic variables with the historical pest occurrence records, our result showed that wind speed, minimum temperature and dew points were the three factors that showed significant correlations in OLS model. The GWR determined correlation coefficients were 0.05, 0.10, 0.12, 0.13, 0.11 and 0.05 for the year 2007, 2008, 2009, 2010, 2011, 2015 and 2016, respectively (Table 1). In most cases, the GWR model showed improved *R*^2^ and AIC value, indicating the presence of spatial relation between the infestation presence and these significant climate factors (Table 2). However, it should be noted that the *R*^2^ values are quite low, indicating that even though there is a spatial relationship the strength of this is quite low. Given the large errors that may be associated with various variables used in such analysis it would be prudent to verify these results with other measures of correlation.

**Table 1:** The results of geographic weight regression (GWR) model with the significant factor/s that resulted from the ordinary least square regression (OLS) model for each year.

Year	Factor/s	*R*^2^
2007	MIN, DEWP	0.05
2008	DEWP	0.10
2010	WDSP, MIN, DEWP	0.12
2011	DEWP	0.13
2012	DEWP	0.11
2016	WDSP, MIN, DEWP	0.05

**Note:**

Min, mean daily minimum temperature; DEWP, mean daily dew points; WDSP, mean daily wind speed.

**Table 2:** *R*^2^ and Akaike’s information criterion (AIC) values of the ordinary least square regression (OLS) and the geographic weight regression (GWR) models.

Year	OLS		GWR	
	*R*^2^	AIC	*R*^2^	AIC
2007	0.05	874.4	0.05	875.0
2008	0.04	890.9	0.10	849.2
2010	0.12	852.9	0.12	846.0
2011	0.05	671.0	0.13	615.3
2012	0.01	918.0	0.11	854.4
2016	0.06	903.0	0.06	903

### Hotspot changes, validation and projections for current climate

The results of the Kriging interpolation indicated annual regional spatial changes of Dubas bug hotspots, of which the majority were in northern Oman (Fig. 2). Our analysis revealed an increased percentage of high risk areas between 2007 and 2009, which decreased between 2010 and 2011. These increased again in 2015 and 2016. The results also revealed that hotspots in north-western Oman by 2010 and 2011 were smaller in size than in the rest of the years analyzed, which could be due to unsuitability of climatic and/or non-climatic factors such as removal offshoots, pesticides and fertilization.

Using the selected Bioclim variables and validation records under maximum sensitivity + specificity threshold, the results indicated that 97% of pest records fell within the high favorability climate categories, as shown in Fig. 2. Projections under current climate show that northern Oman has the most suitable climate while regions between 17–22°N and 52–59°E having an unsuitable climate for Dubas bug occurrence (Fig. 2). Our results also indicated that there are some regions with a marginally suitable climate for the pest (22–23°N and 59–60°E).

### Future projections

**Figure 3:**
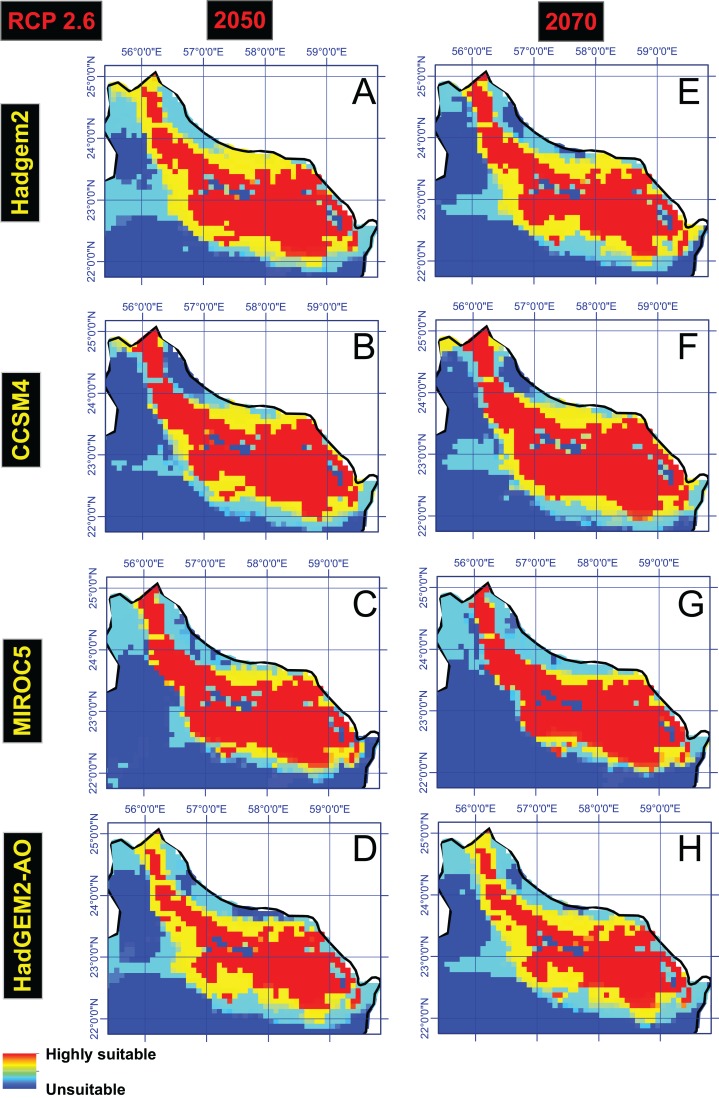
The ensemble model outputs (Climatic suitability) for the Dubas bug using HadGEM2 (A and E), CCSM4 (B and F), MIROC5 (C and G) and HadGEM2-AO (D and H), under RCP of 2.6 for of the years 2050 and 2070.

The future projections obtained from four GCMs, under four RCPs, indicated that northern Oman will remain highly suitable for Dubas bug occurrence and is thus at greater risk. However, all GCMs under all RCPs projected a slight reduction in areas of high suitability class by 2070, compared to 2050. Figure 3 shows the ensemble model outputs (climatic suitability) for the Dubas bug using HadGEM2, CCSM4, MIROC5 and HadGEM2-AO, under RCP of 2.6 for of the years 2050 and 2070; refer to supplementary file for the results of RCP of 4.5, 6.0 and 8.5 under different GCMs.

The future projections of four GCMs, under four RCPs, showed that areas with marginal suitability comprise less than 1% of those areas with the high suitability, but are significantly linked or adjacent to areas of high suitability, indicating a high risk of future Dubas bug infestation.

## Discussion

The historical data analysis showed a significant spatial relation between the infestation occurrence and a few climate factors. However, the results indicated a low correlation coefficient for the relationship, assumed to be due to the use of binary data (presence and absence) in the correlation, which is less powerful than using scale value data (infestation levels). Further, the climate data was estimated using the nearest weather station to the locations, rather than the real climate of the location whereas the tree canopies and variation in intercropping and cultural practices affect the microclimate in each location (Al-Kindi et al., 2017; Al Sarai Al Alawi, 2015; Al Shidi et al., 2018). An earlier investigation showed that temperature had significant effects on the hatching of the Dubas bug eggs (Al-Khatri, 2011). In addition, the author confirmed that temperature variations were associated with variations of infestation and percentage of egg hatching in different locations, seasons and years. A very recent study confirmed the relationship of solar radiation and infestation levels of the Dubas bug (Shidi et al., 2018).

Oman lacks detailed data on irrigation methods, spacing of rows, pruning, removing or retaining suckers, use of insecticides and fertilizers, plantation density per hectare and removing unproductive palms, such that it is difficult to identify reasons for the lower infestation rate between 2010 and 2011.

It is documented that temperature, moisture, windiness and snowfall, amongst other climatic parameters, are major factors influencing the relationships of crops, pests and diseases (Alkishe, Peterson & Samy, 2017; Lonsdale & Gibbs, 1996). Despite the Dubas bugs’ overwhelming impact, there is limited information available on the biogeography of this genus in relation to soil and climate. Although the foremost relationship is between growth rate and temperature, survival ability at extreme temperatures is also of crucial importance (Sangalang, Backhouse & Burgess, 1995).

Our findings indicated that northern Oman is currently at greater risk of Dubas bug infestation and will remain so for 2050 and 2070. Integrating non-climatic pest management methods might complement climatic parameters in monitoring infestation levels in providing information to develop effective strategies to manage Dubas bug infestations in Oman, thus maintaining yield levels and market competitiveness of dates from Oman. However, strategies for adaptation to climate change require creating a link between a stated expectation on how global warming could affect species, habitats or even people and clear objectives and actions that would best address these projected climate changes (Poiani et al., 2011). Conservation effectiveness requires the prioritizing of actions to ensure the continuation of particular species and habitat areas. It is impossible to make conservation interventions for all species and priority decisions should focus on which species to protect. Due to levels of uncertainty and complexity in modeling, altering the prioritizing of certain species before others requires caution (Mesgaran, Cousens & Webber, 2014). In this study, we incorporated the most commonly used thresholds for output classification.

Defining the bioclimatic space in which a species can persist, and regions of continued suitability within this space, may enhance persistence through periods of climatic stress. Correlative, or mechanistic, niche modeling supports the identification of suitable sites. Many previous studies have focused on the direct effects of climate change on particular species. However, the indirect effects within biological communities and alterations to fundamental natural resources may have substantial, complex and even exponential impact on affected species. Thus, it may be the case that current research has not yet mastered the complexity of interactions between invasive species, hosts and the impact of climate change. Further, an ever increasing human population will itself be subjected to the increasing effects of climate change, and the likelihood that human adaptation responses will impact negatively on biodiversity (Watson & Segan, 2013).

Our findings are similar to that of Ferrocino et al. (2013), who concluded that the global warming predicted for the future could induce an increase in the incidence of diseases caused by plant pathogenic species. Governorate of countries projected to become suitable or marginally suitable to the Dubas bug should develop and implement appropriate policies and programs to assure agricultural safety, and develop some form of cash crop biosecurity system. Strict policy in terms of transfer of soil, seed and water from one country or continent to another would be the first necessary step to protect agricultural products, in that there exists a body of literature showing that the disease can spread easily through soil attached to machinery, agricultural tools, water and the movement of plant debris from field to field or within a region or country.

Additional factors possibly affecting date quality include a less experienced, less educated date palm workforce, ideally, a date palm plantation workforce requires training in all aspects of Dubas bug identification and control, including a basic entomological grasp of the life cycles of potential pests. Such training requires funding, or alternatively economic incentives to traditional farming communities to preserve traditional agricultural practices. It should be highlighted that our results mainly estimate the fundamental ecological niche (climatic in nature), which for comparatively small regions may coincide with the distributional area of a species. As we have seen, however, this conclusion is subject to many caveats and conditions (refer to Soberon & Peterson 2005 for more clarification).

We should emphasize that assessments of impacts from anthropogenic climate alteration depend on projections from climate models. Uncertainties in those have often been a limiting issue (Knutti & Sedláček, 2013); additionally they have stated that the previous models on Climate Change Fifth Assessment Report (IPCC AR5) are similar to that from those used in IPCC AR4 after accounting for the different underlying scenarios. However, there differences between models and scenarios are obvious and the uncertainties should not stop decisions being made. In our case, we selected four different GCMs under four different RCPs for three different time periods (current, 2050 and 2070) and our results demonstrated that northern Oman is currently at great risk of Dubas bug infestations (highly suitable climatically) and the potential infestation levels will remain high in 2050 and 2070. We note that we also employed all four available RCPs (2.6, 4.5, 6.0 and 8.5) at a global scale and the results again confirm that northern Oman, the areas under extensive date palm cultivation, will remain at great risk of this pest (see the supplementary file). Thus other non-climatic integrated pest management methods may be of greater value than climatic parameters for monitoring infestation levels. Additionally, the adaptation of both species (host and pest) with climate change is a possible and valid point but impossible to cover.

Generally, successful pest control, particularly major infestations, depends on the support of agricultural extension services. Extension officers, who should themselves be well-trained, have a responsibility to train farmers, particularly in the dangers of continuous use of chemical insecticides, rather than the safer and more efficient alternative agents of control. Further measures could include teams of plant protection officers, with a principal duty to inspect and assess Dubas bug infestations, as well as forwarding unidentified arthropod pests to identification centers and laboratories for the analysis of fungi.

## Conclusion

This research has predicted areas conducive to the Dubas bug under future climate scenarios, as well as the impact on northern Oman of the Dubas bug under projected climate changes. The distribution maps based on the modeling provide a valuable tool for the planning of appropriate agricultural production methods of the future. Relevant information on areas projected as suitable for the pest can be obtained from these maps (Fig. 3 and all figures provided in the supplementary file), that support long-term planning for the management of date palm production and the modeling methods have application for a variety of species.

This paper transfers two messages of “*northern Oman is currently at great risk of Dubas bug infestations and the infestations level will remain high in 2050 and 2070*” and “*Other non-climatic integrated pest management methods may be greater value than climatic parameters for monitoring infestation levels*” to the decision makers.

Factors of significance in the research were: (a) temperature, precipitation and humidity variables were easily accessible; (b) data on the historical distribution of the Dubas bug was available and well-documented; and (c) the four GCMs were selected on the basis of their (i) small horizontal grid spacing and (ii) clarity in representation of current local climate conditions.

Possible study limitations were the unavailability of data on irrigation methods, plantation spacing, pruning and removal or retention of suckers, insecticides and fertilizers, density of trees and the removal of unproductive palms, making it impossible to assess the impact of non-climatic factors on levels of infestation. The modeling undertaken here uses current presence data and future climate projections to project likely future distributions. However, there are many other factors that affect species distributions. The species may also spread to novel climates or adapt in novel climates; something that is not possible to project using current modeling techniques. This is a limitation of the modeling approach used in this research.

In conclusion, our distribution maps will support the expansion of date palm production into areas projected to become unsuitable or marginally suitable for Dubas bug.

## Supplemental Information

10.7717/peerj.5545/supp-1 Supplemental Information 1 RCP 4.5, 6.0 and 8.5 results for Oman and at global scale.

10.7717/peerj.5545/supp-2 Supplemental Information 2 Records of bugs.

## Additional Information and Declarations

### Data Availability

The following information was supplied regarding data availability: The raw data are provided in a Supplemental File.

## References

[ref-1] Abdullah S, Lorca L, Jansson H (2010). Diseases of date palms (*Phoenix dactylifera* L.). Basra Journal for Date Palm Researches.

[ref-2] Al-Khatri S (2011). Biological, ecological and phylogenic studies of Pseudoligosita babylonica viggiani, a native egg parasitoid of Dubas bug Ommatissus lybicus de Bergevin, the major pest of date palm in the Sultanate of Oman.

[ref-3] Al-Kindi KM, Kwan P, Andrew NR, Welch M (2017). Impacts of human-related practices on *Ommatissus lybicus* infestations of date palm in Oman. PLOS ONE.

[ref-4] Alkishe A, Peterson A, Samy A (2017). Climate change influences on the potential geographic distribution of the disease vector tick *Ixodes ricinus*. PLOS ONE.

[ref-5] Allouche O, Tsoar A, Kadmon R (2006). Assessing the accuracy of species distribution models: prevalence, kappa and the true skill statistic (TSS). Journal of Applied Ecology.

[ref-6] Al Sarai Al Alawi M (2015). Studies on the control of Dubas bug, *Ommatissus lybicus DeBergevin* (*Homoptera: Tropiduchidae*), a major pest of date palm in the Sultanate of Oman.

[ref-7] Al Shidi R, Kumar L, Al-Khatri S, Albahri M, Alaufi M (2018). Relationship of date palm tree density to Dubas bug *Ommatissus lybicus* infestation in Omani orchards. Agriculture.

[ref-8] Andrews T, Gregory JM, Webb MJ, Taylor KE (2012). Forcing, feedbacks and climate sensitivity in CMIP5 coupled atmosphere-ocean climate models. Geophysical Research Letters.

[ref-9] Araújo M, New M (2007). Ensemble forecasting of species distributions. Trends in Ecology & Evolution.

[ref-10] Asche M, Wilson M (1989). The plam-feeding planthopper genus Ommatissus (Homoptera: Fulgoroidea: Tropiduchidae). Systematic Entomology.

[ref-11] Baek H, Lee J, Lee H, Hyun Y, Cho C, Kwon W, Marzin C, Gan S, Kim M, Choi D, Lee J, Lee J, Boo K, Kang H, Byun Y (2013). Climate change in the 21st century simulated by HadGEM2-AO under representative concentration pathways. Asia-Pacific Journal of Atmospheric Sciences.

[ref-13] Bellard C, Bertelsmeier C, Leadley P, Thuiller W, Courchamp F (2012). Impacts of climate change on the future of biodiversity. Ecology Letters.

[ref-14] Beyer H (2004). http://www.spatialecology.com/htools/tooldesc.php.

[ref-15] Blumberg D (2008). Review: date palm arthropod pests and their management in Israel. Phytoparasitica.

[ref-16] Brooke C (2008). Conservation and adaptation to climate change. Conservation Biology.

[ref-17] Cantor S, Sun C, Tortolero-Luna G, Richards-Kortum R, Follen M (1999). A comparison of C/B ratios from studies using receiver operating characteristic curve analysis. Journal of Clinical Epidemiology.

[ref-19] Da Silva R, Kumar L, Shabani F, Picanço M (2017). Potential risk levels of invasive *Neoleucinodes elegantalis* (small tomato borer) in areas optimal for open-field *Solanum lycopersicum* (tomato) cultivation in the present and under predicted climate change. Pest Management Science.

[ref-20] Dawson T, Jackson S, House J, Prentice I, Mace G (2011). Beyond predictions: biodiversity conservation in a changing climate. Science.

[ref-21] Dike VN, Shimizu MH, Diallo M, Lin Z, Nwofor OK, Chineke TC (2015). Modelling present and future African climate using CMIP5 scenarios in HadGEM2-ES. International Journal of Climatology.

[ref-22] Dowson V (1936). A serious pest of date palms, *Ommatissus binotatus* Fieb. (Homoptera: Tropiduchidae). Tropical Agriculture.

[ref-23] Elith J, Kearney M, Phillips S (2010). The art of modelling range-shifting species. Methods in Ecology and Evolution.

[ref-24] Elith J, Leathwick J, Hastie T (2008). A working guide to boosted regression trees. Journal of Animal Ecology.

[ref-25] Ferrocino I, Chitarra W, Pugliese M, Gilardi G, Gullino M, Garibaldi A (2013). Effect of elevated atmospheric CO_2_ and temperature on disease severity of *Fusarium oxysporum* f.sp. *lactucae* on lettuce plants. Applied Soil Ecology.

[ref-26] Fitzpatrick M, Weltzin J, Sanders N, Dunn R (2007). The biogeography of prediction error: why does the introduced range of the fire ant over-predict its native range?. Global Ecology and Biogeography.

[ref-27] Fordham D, Bertelsmeier C, Brook B, Early R, Neto D, Brown S, Ollier S, Araújo M (2018). How complex should models be? Comparing correlative and mechanistic range dynamics models. Global Change Biology.

[ref-28] Fotheringham A, Crespo R, Yao J (2015). Geographical and temporal weighted regression (GTWR). Geographical Analysis.

[ref-29] Fourcade Y, Engler J, Rödder D, Secondi J (2014). Mapping species distributions with MAXENT using a geographically biased sample of presence data: a performance assessment of methods for correcting sampling bias. PLOS ONE.

[ref-30] Gassouma M (2004). Pests of the date palm (*Phoenix dactylifera*).

[ref-31] Guisan A, Theurillat J, Kienast F (1998). Predicting the potential distribution of plant species in an alpine environment. Journal of Vegetation Science.

[ref-32] Heffernan O (2010). Earth science: the climate machine. Nature News.

[ref-33] Howard F, Giblin-Davis R, Moore D, Abad R (2001). Insects on palms.

[ref-34] Jiménez-Valverde A (2014). Threshold-dependence as a desirable attribute for discrimination assessment: implications for the evaluation of species distribution models. Biodiversity and Conservation.

[ref-35] Jiménez-Valverde A, Acevedo P, Barbosa A, Lobo J, Real R (2013). Discrimination capacity in species distribution models depends on the representativeness of the environmental domain. Global Ecology and Biogeography.

[ref-36] Kinaway M, Al-Siyabi A (2012). Major arthropod pests of date palm in Arab countries.

[ref-37] Klein M, Venezian A (1985). The dubas date Tropiduchid, Ommatissus binotatus lybicus, a threat to date palms in Israel. Phytoparasitica.

[ref-38] Knutti R, Sedláček J (2013). Robustness and uncertainties in the new CMIP5 climate model projections. Nature Climate Change.

[ref-39] Lawrence D, Oleson K, Flanner M, Fletcher C, Lawrence P, Levis S, Swenson S, Bonan G (2012). The CCSM4 land simulation, 1850–2005: assessment of surface climate and new capabilities. Journal of Climate.

[ref-40] Lobo J, Jiménez-Valverde A, Real R (2008). AUC: a misleading measure of the performance of predictive distribution models. Global Ecology and Biogeography.

[ref-41] Lonsdale D, Gibbs J (1996). Effects of climate change on fungal diseases of trees. British Mycological Society Symposium Series.

[ref-42] Mamoon A, Wright D, Dobson H (2016). Assessing the optimum droplet size for controlling Dubas bug on date palm in the sultanate of Oman when applying an insecticide spray from an aircraft. Outlooks on Pest Management.

[ref-43] Mesgaran M, Cousens R, Webber B (2014). Here be dragons: a tool for quantifying novelty due to covariate range and correlation change when projecting species distribution models. Diversity and Distributions.

[ref-44] Parmesan C (2006). Ecological and evolutionary responses to recent climate change. Annual Review of Ecology, Evolution, and Systematics.

[ref-72] Paterson R, Kumar RM, Shabani F, Lima N (2017). World climate suitability projections to 2050 and 2100 for growing oil palm. The Journal of Agricultural Science.

[ref-45] Pearson R, Dawson T, Berry P, Harrison P (2002). SPECIES: a spatial evaluation of climate impact on the envelope of species. Ecological Modelling.

[ref-46] Peterson A (2006). Uses and requirements of ecological niche models and related distributional models. Biodiversity Informatics.

[ref-47] Peterson A, Campbell L, Moo-Llanes D, Travi B, González C, Ferro M, Ferreira G, Brandão-Filho S, Cupolillo E, Ramsey J (2017). Influences of climate change on the potential distribution of Lutzomyia longipalpis sensu lato (Psychodidae: Phlebotominae). International Journal for Parasitology.

[ref-48] Peterson A, Papeş M, Soberón J (2008). Rethinking receiver operating characteristic analysis applications in ecological niche modeling. Ecological Modelling.

[ref-49] Phillips S, Anderson R, Schapire R (2006). Maximum entropy modeling of species geographic distributions. Ecological Modelling.

[ref-50] Poiani K, Goldman R, Hobson J, Hoekstra J, Nelson K (2011). Redesigning biodiversity conservation projects for climate change: examples from the field. Biodiversity and Conservation.

[ref-51] Ramirez-Cabral NZ, Kumar L, Shabani F (2017). Global risk levels for corn rusts (Puccinia sorghi and Puccinia polysora) under climate change projections. Journal of Phytopathology.

[ref-52] R Development Core Team (2017). R: A Language and Environment for Statistical Computing.

[ref-53] Ridgeway G (2006). https://cran.r-project.org/web/packages/gbm/index.html.

[ref-54] Rosenzweig C, Karoly D, Vicarelli M, Neofotis P, Wu Q, Casassa G, Menzel A, Root TL, Estrella N, Seguin B, Tryjanowski P, Liu C, Rawlins S, Imeson A (2008). Attributing physical and biological impacts to anthropogenic climate change. Nature.

[ref-55] Sangalang A, Backhouse D, Burgess L (1995). Survival and growth in culture of four *Fusarium* species in relation to occurrence in soils from hot climatic regions. Mycological Research.

[ref-56] Shabani F, Kumar L, Ahmadi M (2016). A comparison of absolute performance of different correlative and mechanistic species distribution models in an independent area. Ecology and Evolution.

[ref-57] Shabani F, Kumar L, Ahmadi M (2017). Climate modelling shows increased risk to *Eucalyptus sideroxylon* on the eastern coast of Australia compared to *Eucalyptus albens*. Plants.

[ref-71] Shabani F, Kumar L, Esmaeili A (2015). A modelling implementation of climate change on biodegradation of Low-Density Polyethylene (LDPE) by Aspergillus niger in soil. Global Ecology and Conservation.

[ref-70] Shabani F, Kumar L, Taylor S (2012). Climate change impacts on the future distribution of date palms: a modeling exercise using CLIMEX. PLOS ONE.

[ref-73] Shabani F, Kumar L, Esmaeili A, Saremi H (2013). Climate change will lead to larger areas of Spain being conducive to date palm cultivation. Journal of Food, Agriculture & Environment.

[ref-74] Shabani F, Tehrany MS, Solhjouy-fard S, Kumar L (2018). A comparative modeling study on non-climatic and climatic risk assessment on Asian Tiger Mosquito (Aedes albopictus). PeerJ.

[ref-58] Shidi R, Kumar L, Al-Khatri S, Alaufi M, Albahri M (2018). Does solar radiation affect the distribution of Dubas bug (*Ommatissus lybicus* de Bergevin) Infestation. Agriculture.

[ref-59] Sinervo B, Mendez-De-La-Cruz F, Miles D, Heulin B, Bastiaans E, Villagrán-Santa Cruz M, Lara-Resendiz R, Martínez-Méndez N, Calderón-Espinosa M, Meza-Lázaro R, Gadsden H, Avila LJ, Morando M, De la Riva IJ, Sepulveda PV, Rocha CF, Ibargüengoytía N, Puntriano CA, Massot M, Lepetz V, Oksanen TA, Chapple DG, Bauer AM, Branch WR, Clobert J, Sites JW (2010). Erosion of lizard diversity by climate change and altered thermal niches. Science.

[ref-60] Soberon J, Peterson A (2005). Interpretation of models of fundamental ecological niches and species’ distributional areas. Biodiversity Informatics.

[ref-61] Stocker T, Qin D, Plattner G, Tignor M, Allen S, Boschung J, Nauels A, Xia Y, Bex V, Midgley P (2013). Climate change 2013: the physical science basis.

[ref-62] Taylor KE, Stouffer RJ, Meehl GA (2012). An overview of CMIP5 and the experiment design. Bulletin of the American Meteorological Society.

[ref-63] Thackeray S, Sparks T, Frederiksen M, Burthe S, Bacon P, Bell J, Botham M, Brereton T, Bright P, Carvalho L (2010). Trophic level asynchrony in rates of phenological change for marine, freshwater and terrestrial environments. Global Change Biology.

[ref-64] Thuiller W, Lafourcade B, Engler R, Araújo M (2009). BIOMOD—a platform for ensemble forecasting of species distributions. Ecography.

[ref-65] Urban M (2015). Accelerating extinction risk from climate change. Science.

[ref-66] VanDerWal J, Murphy H, Kutt AS, Perkins G, Bateman B, Perry J, Reside A (2013). Focus on poleward shifts in species’ distribution underestimates the fingerprint of climate change. Nature Climate Change.

[ref-67] Walsh R, Assis A, Paula A, Patton J, Marroig G, Dawson T, Lacey E (2016). Morphological and dietary responses of chipmunks to a century of climate change. Global Change Biology.

[ref-68] Watanabe M, Suzuki T, O’Ishi R, Komuro Y, Watanabe S, Emori S, Takemura T, Chikira M, Ogura T, Sekiguchi M, Takata K, Yamazaki D, Yokohata T, Nozawa T, Hasumi H, Tatebe H, Kimoto M (2010). Improved climate simulation by MIROC5: mean states, variability, and climate sensitivity. Journal of Climate.

[ref-69] Watson J, Segan D (2013). Accommodating the human response for realistic adaptation planning: response to Gillson et al. Trends in Ecology & Evolution.

