# Supplementary material for: Impacts of climate change on infestations of Dubas bug (Ommatissus lybicus Bergevin) on date palms in Oman
Source: PeerJ. 2018 Sep 5;6:e5545. doi: 10.7717/peerj.5545 (PMC6129147; doi:10.7717/peerj.5545)
Supplement: Supplemental Information 1 [file peerj-06-5545-s001.pdf]

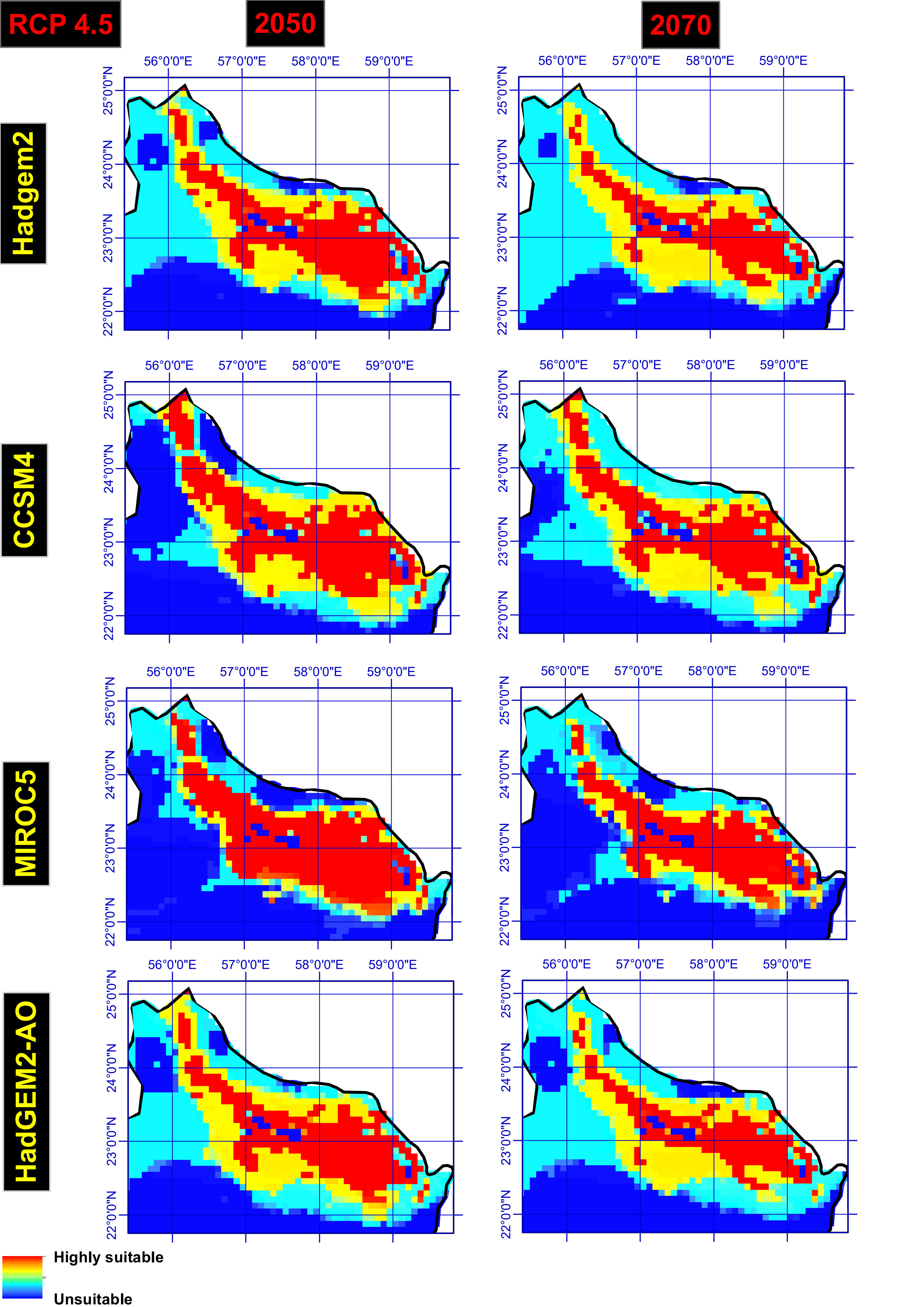

RCP 4.5
2050
2070
Hadgem2
CCSM4
MIROC5
HadGEM2-AO
56°0'0"E
57°0'0"E
58°0'0"E
59°0'0"E
25°0'0"N
24°0'0"N
23°0'0"N
22°0'0"N
Highly suitable
Unsuitable

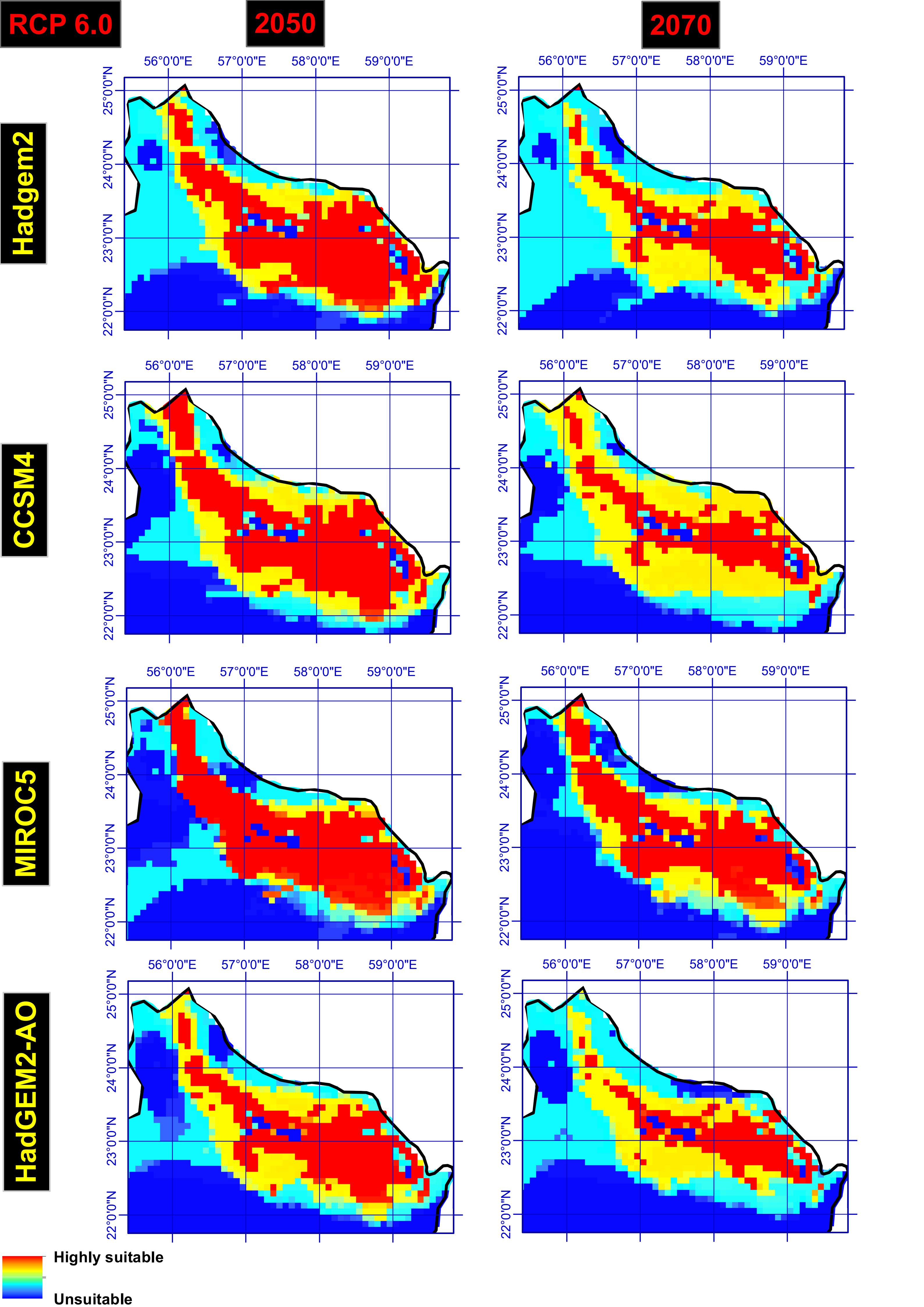
RCP 6.0
2050
2070
Hadgem2
CCSM4
MIROC5
HadGEM2-AO
56°0'0"E
57°0'0"E
58°0'0"E
59°0'0"E
25°0'0"N
24°0'0"N
23°0'0"N
22°0'0"N
Highly suitable
Unsuitable

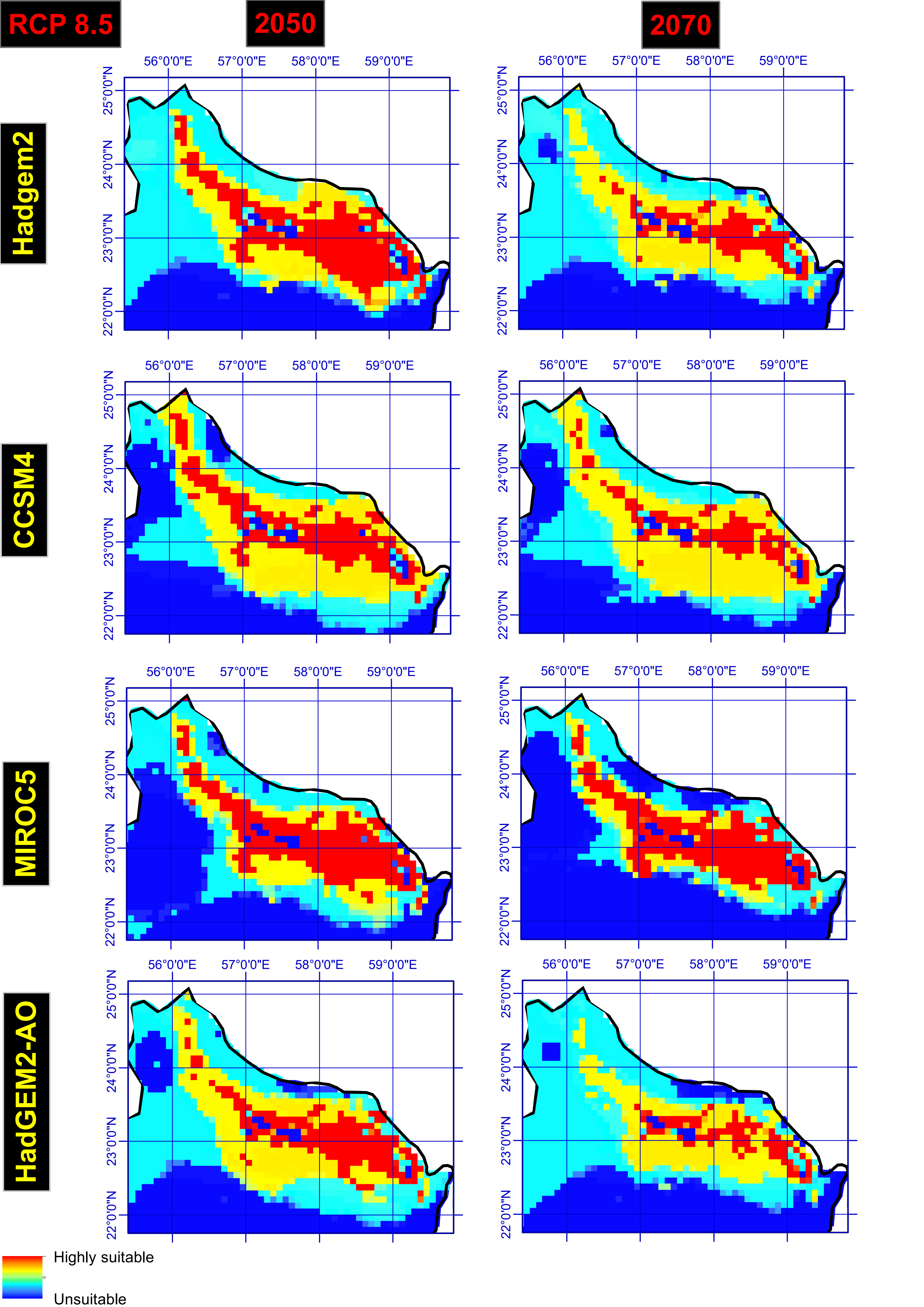
RCP 8.5
2050
2070
Hadgem2
CCSM4
MIROC5
HadGEM2-AO
56°0'0"E
57°0'0"E
58°0'0"E
59°0'0"E
25°0'0"N
24°0'0"N
23°0'0"N
22°0'0"N
Highly suitable
Unsuitable

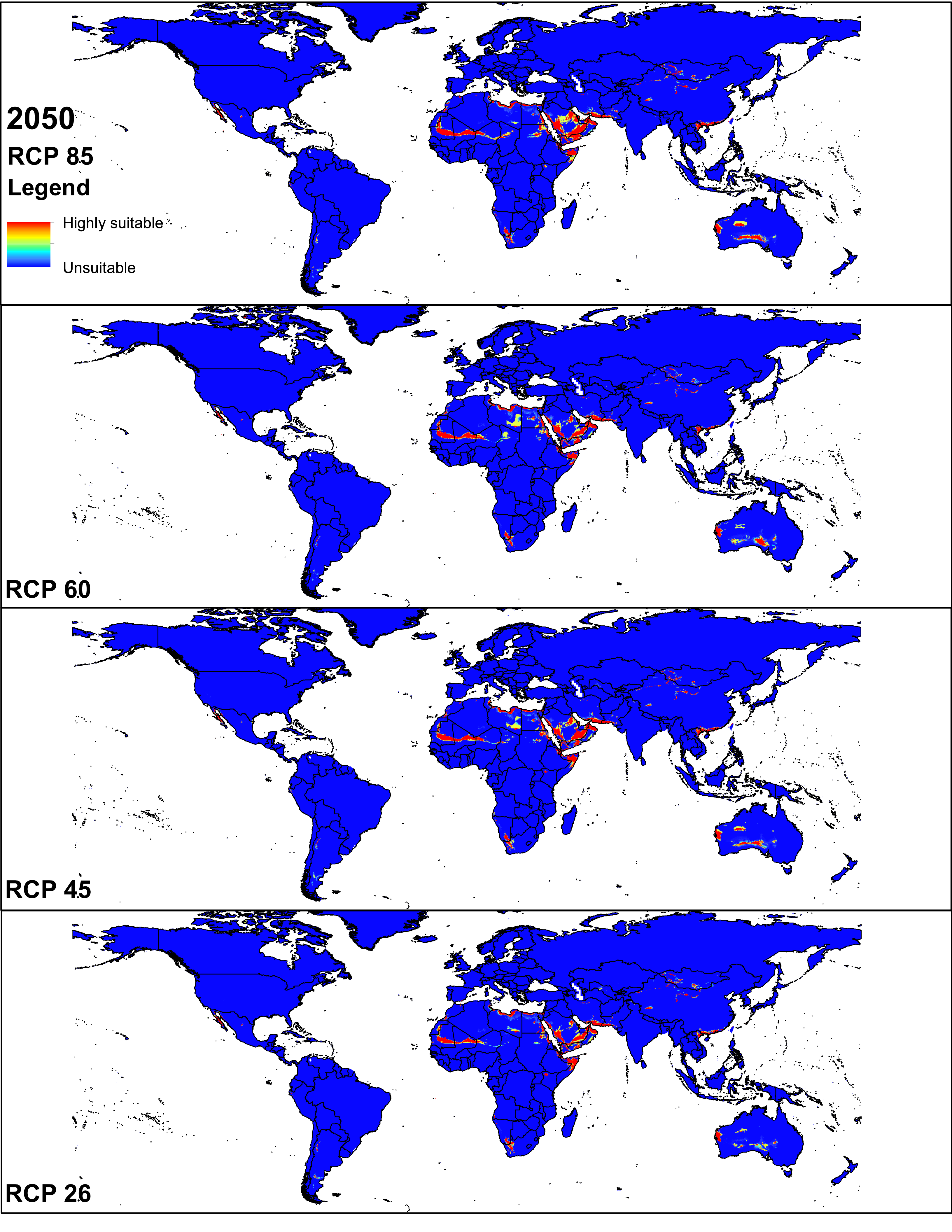
2050
RCP 85
Legend
Highly suitable
Unsuitable
RCP 60
RCP 45
RCP 26

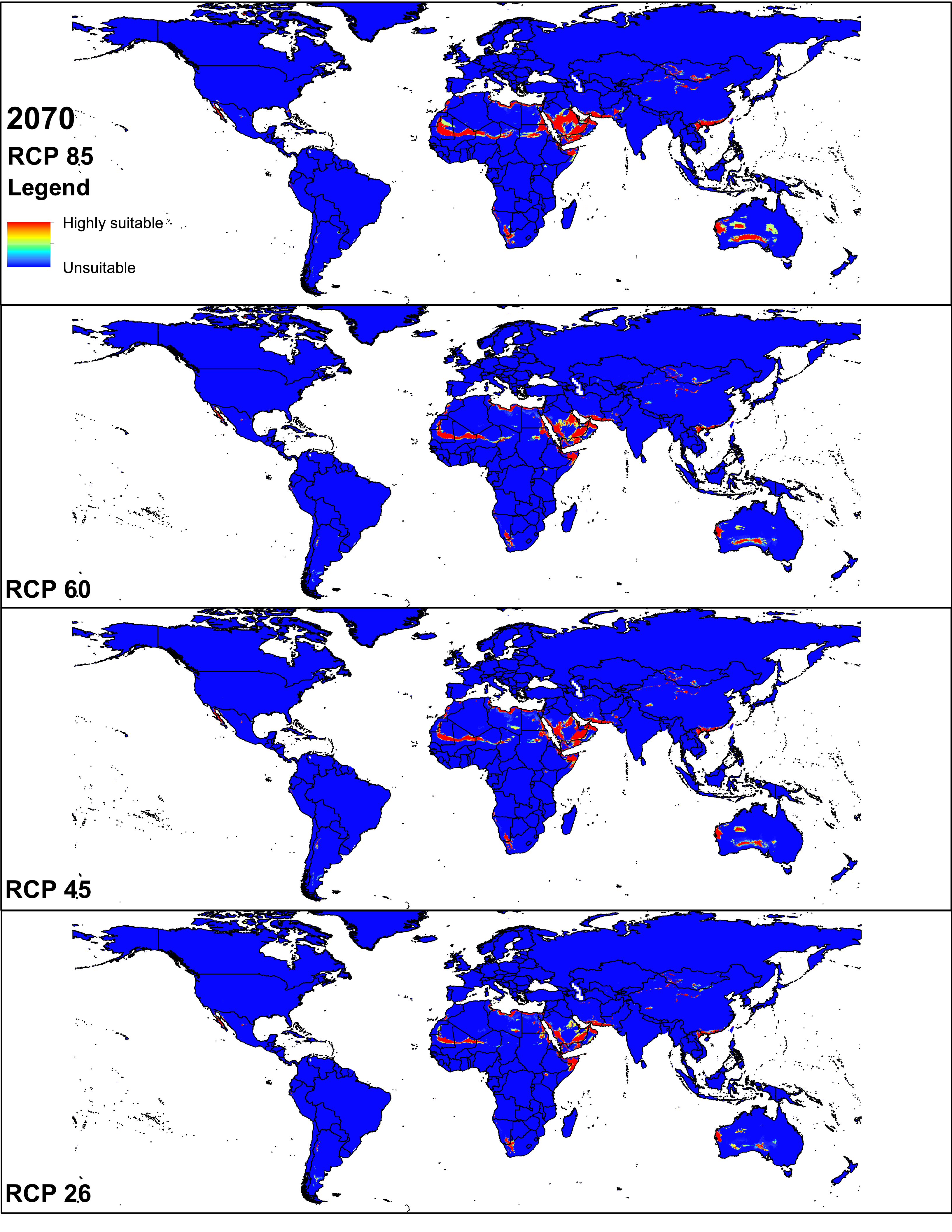
2070
RCP 85
Legend
Highly suitable
Unsuitable
RCP 60
RCP 45
RCP 26
